# Infant Developmental Milestones and Subsequent Cognitive Function

**DOI:** 10.1002/ana.21120

**Published:** 2007-05-08

**Authors:** Graham K Murray, Peter B Jones, Diana Kuh, Marcus Richards

**Affiliations:** 1 Department of Psychiatry, University of CambridgeCambridge; 2 Behavioural and Clinical Neuroscience Institute, University of CambridgeCambridge; 3 Medical Research Council National Survey of Health and Development, Department of Epidemiology and Public Health, University College LondonLondon, United Kingdom

## Abstract

**Objective:**

Developmental delay is associated with a subsequent diagnosis of learning disability. However, the relationship between the age of reaching infant developmental milestones and later intellectual function within the general population remains unresolved. We hypothesized that earlier attainment of developmental milestones would be associated with better subsequent intellectual performance throughout the range of abilities, rather than confined to extremes.

**Methods:**

Developmental data were obtained at age 2 years in the National Survey of Health and Development, a representative sample of 5,362 children born in the United Kingdom in 1946. Data on intellectual function and educational attainment at ages 8, 26, and 53 years were also obtained. Multiple linear regression and logistic regression were used to analyze the effect of age of reaching developmental milestones on subsequent cognition and educational attainment.

**Results:**

The age of reaching developmental milestones was associated with intellectual performance at ages 8, 26, and 53 years; for every month earlier a child learned to stand, there was, on average, a gain of one half of one intelligence quotient point at age 8. Speech development had a small but statistically significant effect on subsequent educational attainment (later developers were less likely to progress beyond basic education); this effect was not apparent for motor development. Effect sizes were reduced when the slowest developers were excluded, but many effects remained significant.

**Interpretation:**

The association between later development and poorer subsequent intellectual function is small, but it does have theoretical implications; we suggest it is secondary to suboptimal cortical-subcortical connectivity. Ann Neurol 2007

Individuals who are markedly late in achieving developmental milestones are at high risk for a subsequent diagnosis of learning disability/mental retardation.^1^, ^2^ However, it has previously been assumed that, within the normal population, there is no relationship between the age of passing developmental milestones and later intellectual function.^3^ Until recently, however, such factors had never been studied in a population sample. Our studies from a population-based sample from Finland have indicated that more rapid infant motor development is linked to better adult neuropsychological performance in some cognitive domains,^4^ to school performance and subsequent educational attainment,^5^ and even to adult brain structure.^6^ Furthermore, such effects are not limited to late developers only as had previously been thought, but rather exist throughout the reference range of development.

The mechanisms underlying infant motor and adult cognitive associations remain poorly characterized. One possibility is that the neural systems that subserve motor development in infancy also contribute to the development and operation of specific cognitive processes later in life. Factors related to efficiencies in such systems may be reflected in both rapid motor development early in life and subsequently in improved cognitive functions.^4^, ^6^ However, a number of questions remain concerning the specificity of associations between infant development and later cognitive functions, which, if they could be answered, could shed light on the reasons behind the associations. For example, is the effect confined to infant motor development, or does it also apply to other developmental domains, such as language? Is the effect confined to specific domains of cognition (eg, executive function), or does it also apply to general intellectual function?

We set out to examine these questions in a large British general population birth cohort in which measurements were available for development in language and motor domains in infancy, general intellectual function in childhood and adolescence, and specific neuropsychological function (eg, verbal fluency, a test of executive/frontal lobe function) in adulthood. We also were interested in whether the association between development and subsequent educational attainment that was demonstrated in Finland would also hold in the United Kingdom.

## Subjects and Methods

### 1946 Birth Cohort

Participants were drawn from the Medical Research Council National Survey of Health and Development. This birth cohort study is stratified by social class and initially consisted of 5,362 people selected from all births that occurred in England, Scotland, and Wales during 1 week in March 1946.^7^ Information about sociodemographic factors and medical, cognitive, and psychological function has been obtained at intervals by interview and examination. The sample is regarded as representative, in most respects, of the UK population born singly and within marriage in the years immediately after the Second World War.^7^

### Developmental Measures, Cognitive Measures, and Educational Attainment

When cohort members were 2 years old, mothers were asked about ages (in months since birth) of standing and walking unaided, the age of saying words other than names for parents, and age of teething. A variety of cognitive tests was used for this analysis. Nonverbal reasoning, reading comprehension (sentence completion), word pronunciation, and vocabulary were examined at age 8.^8^ As adults, participants were tested at age 26 on reading comprehension Watts–Vernon Reading Test (sentence completion)^9^; at age 53, they were tested on verbal fluency (animal naming), which is a test of executive function, and the National Adult Reading Test (NART),^10^ which tests word pronunciation and reflects general intellectual function (for a comparator variable for verbal fluency, an advantage of the NART is that it does not have any executive component). To enable the cognitive scores to be more easily interpretable, we standardized all scores to give scores with a mean of 100 and a standard deviation of 15 (analogous to how intelligence quotient [IQ] scores are usually standardized). IQ scores at age 8 were calculated by summing the verbal and nonverbal scores and restandardizing. The highest educational or training qualification achieved by age 26 was dichotomized either as ordinary secondary qualifications (O levels and their training equivalents) or less, or as advanced secondary education (A levels and their equivalents) or above.^11^

### Statistical Methods

The association between development and cognitive function was investigated with regression models. Separate models were used for each developmental domain. We present models with cognition (at 8, 26, or 53 years) as the dependent variable and development as the independent variable, with adjustment for factors known or considered likely to be determinants of both development and cognitive function: sex, socioeconomic conditions, mother's education, and father's education. Socioeconomic conditions were defined by the British Registrar General's classification of social class and based on father's occupation in childhood and own occupation in adult life. Tests of association between development and cognitive function were repeated with the 5% of the sample who were slowest to develop, and those with IQ scores of less than 70, excluded to check that any statistically significant effect was not driven by those with grossly delayed development. We tested whether relationships between development and cognitive function deviated from linearity by adding a quadratic term (the square of the developmental variable) to the above regression models.^12^ For example, in addition to examining the linear relationship between IQ and age of learning to stand, taking confounding factors into account, we went on to examine the relationship between IQ and (age of learning to stand) *squared*, taking confounding factors into account. As we use developmental precocity as a proxy measure of neural development, we also included a control developmental variable, age of teething, which we predicted would have no relationship to subsequent cognition. Any association between teething and later cognitive function would suggest that maternal recall of development was biased, or that the association between development and cognition was not specifically related to *neuro*development.

In additional analyses, to test whether *change* in cognitive function between childhood and adulthood differed with developmental precocity, models with the cognitive score at age 26 or 53 as the outcome were further adjusted for IQ at age 8.

The effect of development on educational qualifications was assessed by logistic regression, with adjustment for the same confounders as for the cognitive function models. All statistical analysis was conducted using Intercooled Stata 8.0 (Stata Corporation, College Station, TX).

## Results

### Missing Data

Information on at least one cognitive score (IQ at age 8, reading comprehension at age 26, or verbal fluency or NART at age 53) was available for 4,709 subjects. Of these, 3,969 (84%) subjects had information on at least one developmental variable in addition to complete information on all confounders, and could therefore be included in the analysis. Those with missing information did not differ from those with complete information in IQ at age 8 (*p* = 0.8; n = 4,256), but they had higher reading comprehension scores at 26 (*p* < 0.0001; n = 3,714) and verbal fluency scores at age 53 (*p* = 0.056; n = 2,949).

### Infant Development and Subsequent Cognitive Function

#### EFFECTS OF INFANT MOTOR AND SPEECH DEVELOPMENT ON SUBSEQUENT COGNITION: LINEAR FITS

**Fig. 1:**
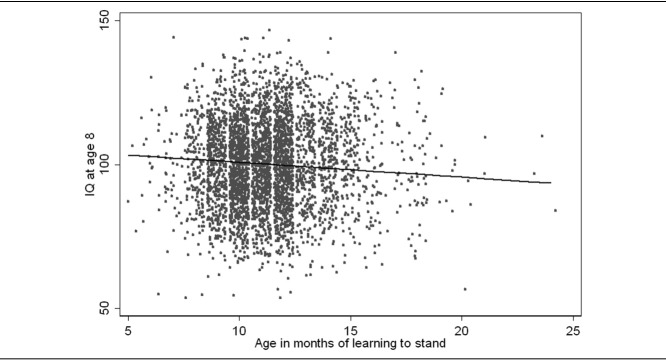
Intelligence quotient (IQ) at age 8 in relation to age at learning to stand in entire sample: linear regression adjusted for sex, socioeconomic conditions, maternal education, and paternal education.

Linear modeling demonstrated that earlier motor development and development of speech were significantly associated with greater IQ at age 8, higher reading comprehension at age 26, and better performance on verbal fluency at age 53 after adjustment for confounders (Table 1, Fig 1). Regression coefficients indicate that the effect of development on later cognition is small. For example, for every month earlier an individual learned to stand, there would be, on average, a gain of one half of one IQ point at age 8. Earlier development in speech and motor domains was also associated with better reading comprehension at age 26. In contrast, there was no association between age of teething and later cognitive function at any age. There was no association between development and NART score at age 53.

**Table 1:** Results of Regression Analyses: Childhood Intelligence Quotient, Reading Comprehension at Age 26, Verbal Fluency and National Adult Reading Test at Age 53 Regressed on Developmental Variables with Sex, Socioeconomic Conditions, Maternal Education, and Paternal Education Included in the Model as Covariates

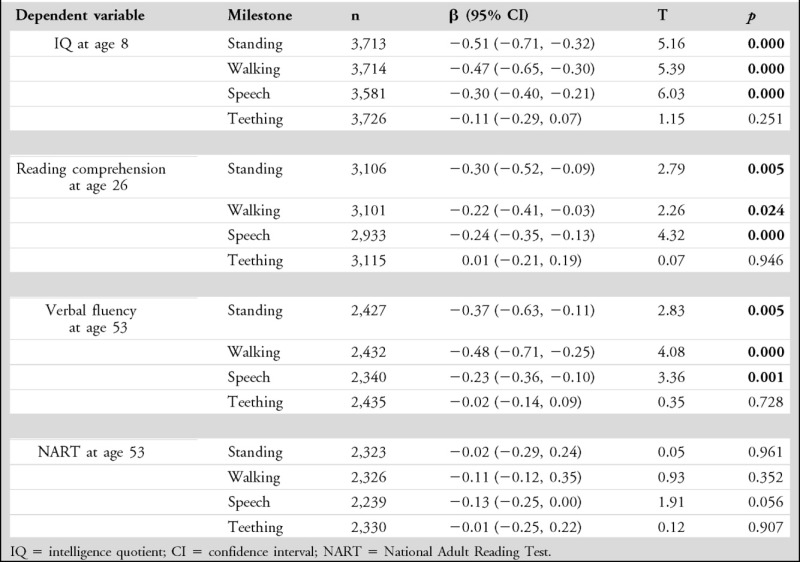

IQ = intelligence quotient; CI = confidence interval; NART = National Adult Reading Test.

#### ANALYSES RESTRICTED TO THE REFERENCE RANGE OF DEVELOPMENT AND INTELLIGENCE QUOTIENT

**Fig. 2:**
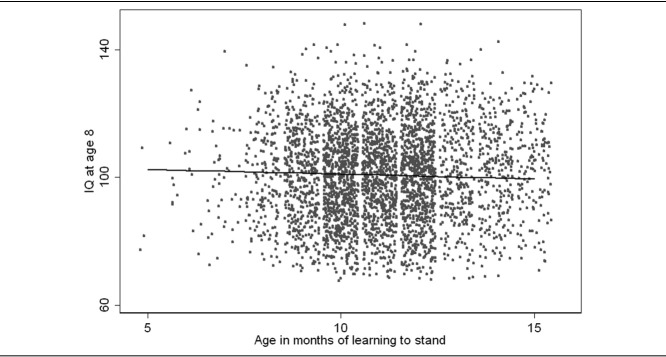
Intelligence quotient (IQ) at age 8 in relation to age at learning to stand restricted to reference range: linear regression adjusted for sex, socioeconomic conditions, maternal education, and paternal education.

To check that the associations between development and cognition were not purely being driven by the latest developers, we repeated the analyses having excluded the slowest 5% of developers and subjects scoring within the learning disability range on IQ tests (IQ < 70; Table 2). The associations between age at first standing and talking and IQ at age 8 were attenuated, but they remained statistically significant (standing: *p* = 0.02, Fig 2; speech: *p* = 0.001). Thus, within the reference range of development, for each month earlier a child learned to stand there was a corresponding 0.3 point IQ gain at age 8. The associations between speech development and reading comprehension at age 26 (*p* = 0.005) and verbal fluency at age 53 (*p* = 0.017) also remained significant. However, the associations between infant motor development and cognition at ages 26 and 53 were no longer statistically significant with the slowest developers removed from the analyses.

**Table 2:** Results of Regression Analyses Restricted to “Normal Developers”: Childhood Intelligence Quotient, Reading Comprehension at 26, Verbal Fluency and National Adult Reading Test at Age 53 Regressed on Developmental Variables with Sex, Socioeconomic Conditions, Maternal Education, and Paternal Education Included in the Model as Covariates

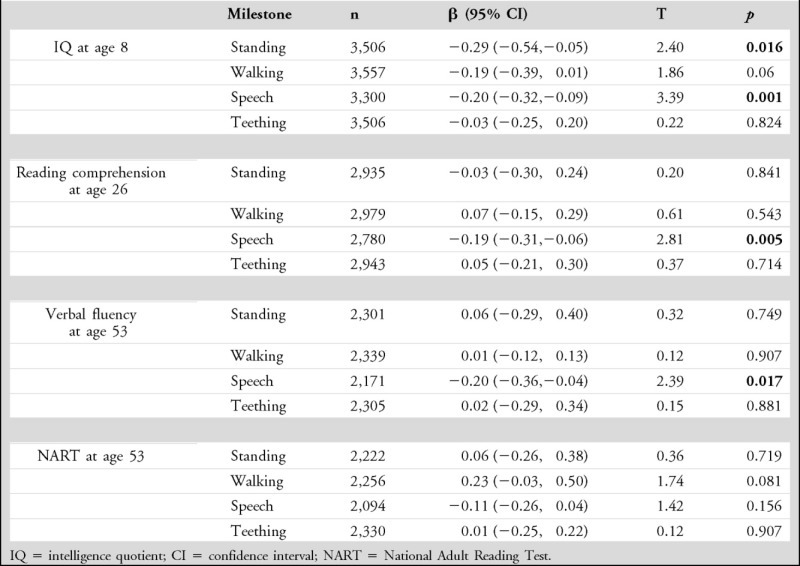

IQ = intelligence quotient; CI = confidence interval; NART = National Adult Reading Test.

#### EFFECTS OF INFANT MOTOR AND SPEECH DEVELOPMENT ON SUBSEQUENT COGNITION: QUADRATIC REGRESSION MODELS

**Fig. 3:**
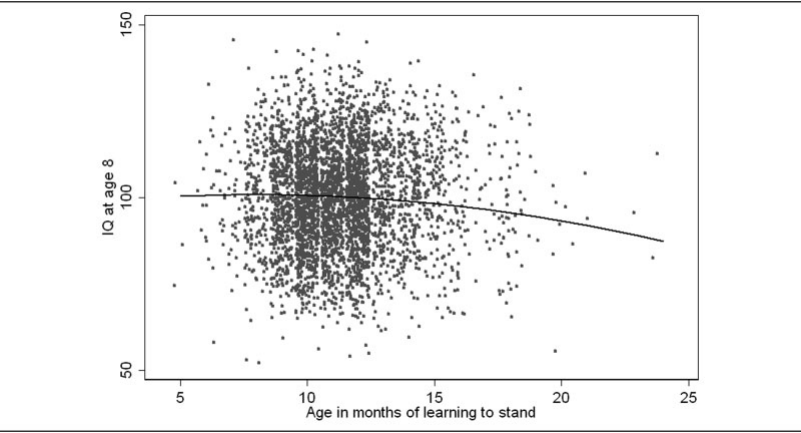
Intelligence quotient (IQ) at age 8 in relation to age at learning to stand in entire sample: quadratic regression adjusted for sex, socioeconomic conditions, maternal education, and paternal education.

After examining linear relationships between IQ and development, we tested for deviation from linearity by adding a quadratic term in the regression. Using the full range of development, we found significant quadratic fits between cognitive outcomes and developmental variables (Table 3; illustrated for standing in Fig 3). However, for the reference range of development and normal IQ, there was no significant quadratic relationship between standing and IQ at age 8 (Table 4). A quadratic fit remained significant for relationships between speech development and cognitive measures at ages 8 and 26, though not for verbal fluency at age 53 (see Table 4).

**Table 3:** Quadratic Regression Analyses: Childhood Intelligence Quotient, Reading Comprehension at 26, Verbal Fluency and National Adult Reading Test at Age 53 Regressed on Developmental Variables with Sex, Socioeconomic Conditions, Maternal Education, and Paternal Education Included in the Model as Covariates

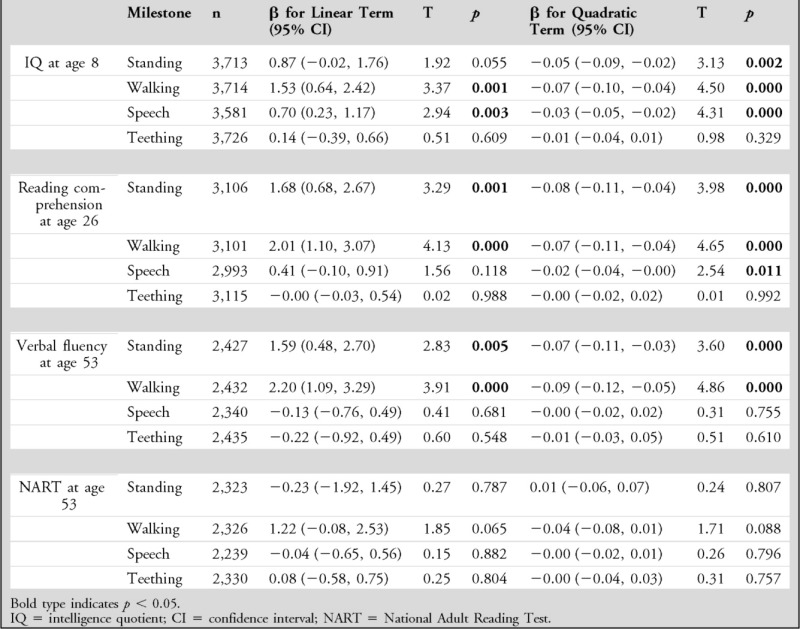

Bold type indicates *p* < 0.05.

IQ = intelligence quotient; CI = confidence interval; NART = National Adult Reading Test.

**Table 4:** Quadratic Regression Analyses Restricted to “Normal Developers”: Childhood Intelligence Quotient, Reading Comprehension at Age 26, Verbal Fluency and National Adult Reading Test at Age 53 Regressed on Developmental Variables with Sex, Socioeconomic Conditions, Maternal Education, and Paternal Education Included in the Model as Covariates

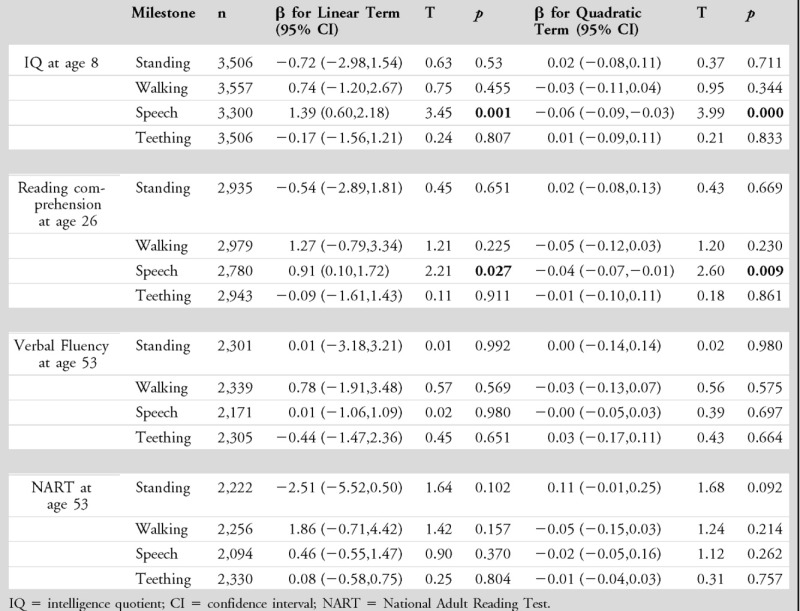

IQ = intelligence quotient; CI = confidence interval; NART = National Adult Reading Test.

#### EFFECT OF DEVELOPMENT ON CHANGE IN INTELLIGENCE QUOTIENT AFTER AGE 8

The majority of the associations between developmental data and cognitive measures after age 8 were shown to be reliant on the earlier relationships between development and IQ at 8, because they became nonsignificant when IQ at 8 was added into the regression models as an independent variable (not shown). The only associations between development and cognition that remained significant after taking into account IQ at age 8 were the quadratic associations in the entire sample between age at standing and reading comprehension at age 26 and between age at first walking and reading comprehension at age 26.

### Infant Development and Educational Outcome

In this sample, 67% of the sample had ordinary secondary level qualifications or less and 33% had advanced secondary level qualifications or above. For motor developmental domains and teething (adjusted for confounders), there was no relationship between developmental and later educational attainment (standing: *p* = 0.9; walking: *p* = 0.3; teething: *p* = 0.6). There was a statistically significant association between speech development and educational attainment (*p* = 0.004), the later developers were less likely to progress to “A” levels; however, the effect is small (odds ratio, 0.97; confidence interval, 0.96–0.99). Thus, for each month later that an infant developed speech, the odds of progressing to “A” levels were reduced by 3% (after adjustment). The effect was only slightly attenuated when the slowest 5% of infants were excluded from the analysis (OR, 0.976); thus, within the reference range of speech development, a 2-month difference in age of speaking equated to a 5% difference in odds of progressing beyond basic education. The effect was no longer significant when IQ at age 8 was added into the model as an independent variable.

## Discussion

In this study, we have shown, in a sample representative of the general population, that infant development is associated with subsequent general intellectual function. Importantly, the effect is not solely driven by late developers, but applies within the reference range of development, albeit to a slightly reduced degree. This extends our previous finding from the Northern Finland 1966 Birth Cohort that faster attainment of motor developmental milestones is related to better adult cognitive performance in some domains, such as executive function, but not others, such as visual learning.^4^

To date, there has been little population-based research into the cognitive sequelae of variations in the speed of development, but there are some prior studies in smaller, nonepidemiological samples that yielded results consistent with our findings. Bayley^13^ studied a sample of 63 subjects followed up from just after birth to 36 years; speech development was significantly correlated with subsequent IQ in girls but not in boys. In one study of 69 school-aged children seen at a pediatrics clinic in Ohio, retrospectively collected information on developmental milestones explained 34 to 43% of the variance in subsequent IQ. However, this relationship was considerably weakened when sociodemographic variables and medical diagnosis were taken into account.^14^ Capute and colleagues^15^ found that age of learning to walk was significantly associated with IQ at age 3 in a primary care cohort of 213 upper-middle-class children; 4 to 6% of the variance in IQ could be explained with respect to motor development.^15^

In this study, we have demonstrated that the relationship between infant development and subsequent cognitive function is not specific to the motor domain but also extends to speech development. Indeed, in this sample, the consequences of variations in the speed of speech development appeared to be more persistent than the consequences of motor developmental variation. Speech development was related to reading comprehension at age 26 even when late developers were excluded, whereas the motor development effect on reading comprehension in adulthood was no longer statistically significant when the analysis was restricted to the reference range of development.

Why should the timing of speech development have a greater effect on reading comprehension in adulthood in comparison with the timing of motor development? Measures of language comprehension at 25 months are related to faster and more accelerated growth in expressive vocabulary across the second year,^16^ indicating that comprehension is intricately linked to speech development. It may be that our finding relates to the specific nature of the cognitive measure at age 26, and reflects continuity in performance in this domain over time. As the only cognitive assessment at age 26 was reading comprehension, we are unable to test whether the same finding would also obtain general intellectual measures or other specific cognitive measures.

We have previously speculated that the mechanisms behind these long-range associations may reflect common neural substrates for different motor and cognitive functions at varying stages of development. Rapid maturation of basic neural circuits involved in the attainment of developmental milestones may also lead to favorable development of more complex cortical-subcortical circuits involved in higher cognitive process later in life.^4^ Prior studies indicate that early connectivity between brain regions imparts sustained, mutually trophic, or protective effects to such regions.^17^ Thus, early establishment of functional connectivity between the frontal cortex and basal ganglia may lead not only to early development of motor skills, but also, through such trophic effects, to improved frontal and subcortical structure. In consequence, there may be improved function in later childhood and, indeed, adulthood. Recent evidence that adult brain volume in the frontal cortex, basal ganglia, and cerebellum is linearly related to the speed of infant motor development provides some support for this hypothesis.^6^

Motor and speech development in infancy rely not only on specific processes but also on more general psychological functions such as response selection, behavioral adaptation, and categorization,^18^–^21^ which are also critical for “executive” cognitive functions later in life. It is notable that at age 53, development was associated with verbal fluency but not NART score, suggesting a stronger relationship exists between development and executive function than between development and general intellectual function. We did find a relationship between development and general intellectual function at age 8, but IQ scores are not independent of executive function, so this is not surprising. For example, it has been claimed that the construct of general intellectual function is subserved by a region of the lateral frontal cortex important in the control of diverse forms of behavior.^22^

We have partially replicated our previous finding that infant development is linked to subsequent educational attainment. However, in this sample, it was in speech development, not motor development (as had been apparent in the 1966 Northern Finland Birth Cohort), that this association was manifest. There are a number of possible reasons for this; for example, there are differences in the two cohorts in timing of developmental assessments, social context, and educational opportunities. Opportunities for children born in comparatively egalitarian Finland 1966 were different from those born in 1946 in the United Kingdom. In the 1946 UK sample, progression to higher education was much less common than in people born in Northern Finland in 1966. In the Finnish sample, 40% went on to tertiary education, compared with less than 10% in this study, which would have reduced the likelihood of the detection of an effect in this study. This may explain the lack of association between motor development and educational attainment in this sample, but it does not explain why an effect in the speech domain was present, which suggests that early development of speech may be more important than motor development in regard to subsequent educational attainment.

Previous studies of the cognitive sequelae of developmental variance across the life span have tended to be drawn from nonrepresentative samples or specific populations such as low birth weight or low IQ. Our cohort is a nationally representative sample of men and women born in Britain in 1946. The design of this study provides a number of other advantages: the subjects are all the same age, eliminating confounding from this factor. The fact that the data were collected at a time contemporaneous with developmental milestones is likely to have resulted in increased accuracy of measurement. A potential criticism is that the developmental data were collected by interviews with mothers as opposed to repeat examinations for research purposes by a pediatrician using validated developmental scales. However, any putative measurement error in developmental measures would be likely to obscure a signal and lead to type II error as opposed to type I error. In future studies, if more precise measurements of neurodevelopment are available, larger effects might be demonstrated. A strength of the study is the inclusion of a comparison developmental measure: teething. This was not related to the development of the nervous system in the same way as speech and motor development, and thus acted as a control factor with respect to maternal recall. The lack of effects with this control measure provides evidence that the results are not generalizable to all developmental domains, but have some specificity to neurodevelopmental domains.

The effects we have demonstrated are small but are important for theoretical reasons and could have public health implications. Variations in the speed of infant development are related to subsequent psychiatric disorders such as depression^23^, ^24^ and schizophrenia,^25^ and have recently been shown to relate to midlife physical performance, which is itself a predictor of subsequent disability and mortality.^26^ Lower childhood and adolescent IQ and lower educational attainment are associated with increased rates of subsequent cardiovascular disease, hypertension, suicide, schizophrenia and other psychiatric disorders, and indeed all-cause mortality.^27^–^33^ Lower cognitive functioning in adulthood is also a predictor of morbidity and mortality.^30^, ^34^, ^35^ Enhanced understanding of the determinants and consequences of normal neural, motor, and cognitive development is important to contextualize research into a variety of disorders that involve subtle disturbances of the normal trajectory of brain development.

## References

[1] Schillace R (1964). Developmental comparisons of mentally retarded and neurotic children. Am J Ment Defic.

[2] von Wendt L, Makinen H, Rantakallio P (1984). Psychomotor development in the first year and mental retardation—a prospective study. J Ment Defic Res.

[3] Eliot L (2001). Early intelligence. How the brain and mind develop in the first five years of life.

[4] Murray GK, Veijola J, Moilanen K (2006). Infant motor development is associated with adult cognitive categorisation in a longitudinal birth cohort study. J Child Psychol Psychiatry.

[5] Taanila A, Murray GK, Jokelainen J (2005). Infant developmental milestones: a 31-year follow-up. Dev Med Child Neurol.

[6] Ridler K, Veijola JM, Tanskanen P (2006). Fronto-cerebellar systems are associated with infant motor and adult executive functions in healthy adults but not in schizophrenia. Proc Natl Acad Sci U S A.

[7] Wadsworth ME, Butterworth SL, Hardy RJ (2003). The life course prospective design: an example of benefits and problems associated with study longevity. Soc Sci Med.

[8] Pigeon DA, Douglas JWB (1964). Tests used in the 1954 and 1957 surveys. The home and the school.

[9] Pigeon DA, Douglas JWB, Ross JM, Simpson HR (1968). Details of the fifteen years tests. All our future.

[10] Nelson HE (1982). The national adult reading test (NART).

[11] Richards M, Hardy R, Kuh D, Wadsworth ME (2001). Birth weight and cognitive function in the British 1946 birth cohort: longitudinal population based study. BMJ.

[12] Clayton D, Hillis M (1993). Statistical methods in epidemiology.

[13] Bayley N (1968). Behavioral correlates of mental growth: birth to 36 years. Am Psychol.

[14] Funk JB, Ruppert ES, Jurs S (1982). Assessing the predictive validity of developmental milestones. J Dev Behav Pediatr.

[15] Capute AJ, Shapiro BK, Palmer FB (1985). Cognitive-motor interactions: the relationship of infant gross motor attainment to IQ at 3 years. Clin Pediatr.

[16] Fernald A, Perfors A, Marchman VA (2006). Picking up speed in understanding: speech processing efficiency and vocabulary growth across the 2nd year. Dev Psychol.

[17] Sherrard RM, Bower AJ (1998). Role of afferents in the development and cell survival of the vertebrate nervous system. Clin Exp Pharmacol Physiol.

[18] Kuhl PK (1993). Developmental speech perception: implications for models of language impairment. Ann NY Acad Sci.

[19] Thelen E (1995). Motor development: a new synthesis. Am Psychol.

[20] Kuhl PK (2004). Early language acquisition: cracking the speech code. Nat Rev Neurosci.

[21] Kuhl PK, Tsao FM, Liu HM (2003). Foreign-language experience in infancy: effects of short-term exposure and social interaction on phonetic learning. Proc Natl Acad Sci U S A.

[22] Duncan J, Seitz RJ, Kolodny J (2000). A neural basis for general intelligence. Science.

[23] van Os J, Jones P, Lewis G (1997). Developmental precursors of affective illness in a general population birth cohort. Arch Gen Psychiatry.

[24] Colman I, Croudace TJ, Ploubidis GB (2005). Childhood developmental predictors of mental health during a forty-year period from adolescence to adulthood. Pediatric Research.

[25] Jones P, Rodgers B, Murray R, Marmot M (1994). Child development risk factors for adult schizophrenia in the British 1946 birth cohort. Lancet.

[26] Kuh D, Hardy R, Butterworth S (2006). Developmental origins of midlife physical performance: evidence from a British birth cohort. Am J Epidemiol.

[27] Zammit S, Allebeck P, David AS (2004). A longitudinal study of premorbid IQ Score and risk of developing schizophrenia, bipolar disorder, severe depression, and other nonaffective psychoses. Arch Gen Psychiatry.

[28] Batty GD, Deary IJ (2004). Early life intelligence and adult health. BMJ.

[29] Batty GD, Deary IJ (2005). Education and mortality: the role of intelligence. Lancet.

[30] Richards M, Deary IJ (2005). A life course approach to cognitive reserve: a model for cognitive aging and development?. Ann Neurol.

[31] Gunnell D, Magnusson PK, Rasmussen F (2005). Low intelligence test scores in 18 year old men and risk of suicide: cohort study. BMJ.

[32] Huisman M, Kunst A, Deeg D (2005). Educational inequalities in the prevalence and incidence of disability in Italy and the Netherlands were observed. J Clin Epidemiol.

[33] Huisman M, Kunst AE, Bopp M (2005). Educational inequalities in cause-specific mortality in middle-aged and older men and women in eight western European populations. Lancet.

[34] Smits CH, Deeg DJ, Kriegsman DM, Schmand B (1999). Cognitive functioning and health as determinants of mortality in an older population. Am J Epidemiol.

[35] McGuire LC, Ford ES, Ajani UA (2006). Cognitive functioning as a predictor of functional disability in later life. Am J Geriatr Psychiatry.

